# Supplementary material for: Examining the role of information integration in the continued influence effect using an event segmentation approach
Source: PLoS One. 2022 Jul 18;17(7):e0271566. doi: 10.1371/journal.pone.0271566 (PMC9292086; doi:10.1371/journal.pone.0271566)
Supplement: S1 File — (DOCX) [file pone.0271566.s001.docx]

**Supplement**

**Experiment 1**

**Event Reports**

**Scenario 1**

***Part 1—Airplane Landing.***

*Message 1*. Passengers on a commercial flight en route to Los Angeles received a terrible fright yesterday as their plane required an emergency landing.

*Message 2*. Peter Faulkner, the pilot of the aircraft, made the decision to land after he was having difficulties controlling the plane.

*Message 3*. The pilot initiated emergency protocol and the aircraft began its descent two hours earlier than it was scheduled to land.

*Message 4* *[Misinformation]*. The Federal Aviation Administration believes the pilot made the right decision, and attributed difficulties controlling the aircraft to an electrical fault caused by the extreme weather conditions he was flying in.

*Message 5*. The aircraft was able to make a safe landing at Portland International airport, and all 540 passengers on board were evacuated without problem.

*Message 6*. Emergency crews were on standby. Passengers were taken to a waiting area where ambulance officers treated 56 passengers who sustained minor injuries.

***Part 2—Airplane Landing.***

*Message 7*. Passengers on a commercial flight that had to make an emergency landing at Portland International airport were forced to stay overnight while the airline arranged a suitable replacement aircraft.

*Message 8*. Marie Scott, a passenger on the flight, told reporters of her ordeal. “It was horrible, all loose items were getting thrown around the cabin, and the seatbelt was hardly containing me. I’m glad I’m safe, but I just really want to go home to my family now.”

*Message 9*. The pilot and crew have been praised for their quick thinking and response. One crew member stated “This is what we’ve been trained for”.

*Message 10* *[Retraction]*. Meanwhile, air crash investigators have found that previous attributions of the emergency landing were incorrect, and that the difficulty controlling the airplane stemmed from physical deterioration of the aircraft’s rudder system.

*Message 10* *[Control]*. The airline released a statement saying, “Safety is our number 1 priority” and apologised to passengers for the inconvenience.

*Message 11*. The aircraft involved was an A380 Airbus, the largest passenger airplane in the world.

*Message 12*. The landing was further complicated by the fact that the only runway in Portland long and wide enough to accommodate the aircraft was temporarily closed for minor maintenance works.

**Scenario 2**

***Part 1—Bushfire.***

*Message 1*. Firefighters in Victoria have been battling a bushfire that raged out of control in the state’s North-East overnight.

*Message 2*. The bushfire came dangerously close to homes in the town of Euroa, but it is believed that no damage was caused to property.

*Message 3*. Due to thick smoke and poor visibility, surrounding roads have been closed. Police have set up road closures, and traffic detours are in place.

*Message 4* *[Misinformation]*. David Karle of the Country Fire Authority (CFA) indicated that authorities were looking into the cause of the fire, with available evidence suggesting that the fire had been deliberately lit.

*Message 5*. Emergency services were still working tirelessly this morning to extinguish the flames, but were confident that the location of the remaining fire was unlikely to pose any further threat to local communities.

*Message 6*. The fire has been especially difficult to control due to the hot, dry weather conditions.

***Part 2—Bushfire.***

*Message 7*. After working throughout the day, firefighters have managed to bring a bushfire in the North-East of Victoria under control.

*Message 8*. There have been no reported casualties or damage to property, with most land damage occurring in rural fringe areas and nearby forest reserves. The suspected burn area is estimated to be roughly 50,000 hectares.

*Message 9*. Fire captain, Fred Davis, praised his fire fighters for their tireless work under difficult circumstances.

*Message 10* *[Retraction]*. After a full investigation, authorities have concluded that original reports were incorrect, and that the fire was set off by lightning strikes.

*Message 10* *[Control]*. Surrounding roads have been re-opened and detours are no longer in place.

*Message 11*. Casey Haas, a resident of Euroa, expressed her relief that no one had been harmed by the fire, and said she felt lucky that they had avoided disaster.

*Message 12*. Casey went on to say she feels it is important for residents of the community to work together to ensure they are prepared for disaster if it ever strikes again.

**Scenario 3**

***Part 1—Water Source.***

*Message 1*. The Freemont Water Department was forced to shut down its water intake from its main water supply, the Denroy River, due to contamination concerns following reports of large scale fish deaths in the waterway.

*Message 2*. The water department supplies water to the Shelby region, including the towns of Monroe, Westphalia, and Freemont itself.

*Message 3*. Water restrictions have been put in place for the Shelby area. Residents have expressed concerns as to the length of time until they can return to normal water usage.

*Message 4* *[Misinformation]*. It is believed that the fish deaths are due to chemical waste dumping by a riverside pharmaceutical company, in violation of the Missouri Clean Water Act.

*Message 5*. A spokesperson of the water department apologised to customers for the inconvenience and emphasised that the department was working tirelessly to sort out the issue.

*Message 6*. Freemont mayor, Kate Gettys, explained that the department remained committed to ensuring that customers can be confident that their water supply is of the purest quality.

***Part 2—Water Source.***

*Message 7*. The Freemont water department has been given the all clear to continue water intake from the Denroy River, after operations ceased for 5 days due to large scale fish deaths in the waterway.

*Message 8*. Residents are relieved that water restrictions have now been lifted.

*Message 9*. The department has begun researching alternative water supplies to reduce its reliance on the river intake in the future.

*Message 10* *[Retraction]*. Tests have revealed that previous suspicions regarding the cause of the fish deaths were unfounded. The tests found that the deaths were due to a rare fish ailment that presents no harm to humans.

*Message 10* *[Control]*. Alternative water supplies will require alterations to the Freemont water department’s infrastructure.

*Message 11*. The shutdown was bad news for the Freemont water department, as recent drought periods had resulted in record low storage levels.

*Message 12*. Despite these ongoing concerns, a spokesperson has assured customers that the local drinking water is as safe as it has ever been.

**Scenario 4**

***Part 1—Nightclub.***

*Message 1*. A woman has been taken to hospital after losing consciousness while out partying at the Cable nightclub in London last night.

*Message 2*. After noticing that she was in trouble, her friends decided to call an ambulance, which took her to St. Mary’s hospital.

*Message 3*. A friend of the woman said she had complained of hallucinations and nausea not long before falling unconscious. The woman herself has no memory of what happened.

*Message 4* *[Misinformation]*. Doctors believe the young lady’s symptoms are the result of her drink getting spiked.

*Message 5*. Cable nightclub is a popular venue with approximately 1,500 patrons visiting it each weekend.

*Message 6*. The nightclub recently re-opened after being closed for twelve months for upgrades and renovations.

***Part 2—Nightclub.***

*Message 7*. A young lady who fell unconscious while partying at a nightclub has remained in hospital.

*Message 8*. The lady was out celebrating with friends after graduating from The Regent Academy, where the group had studied photography together.

*Message 9*. When she lost consciousness, it was the timely aid of her friends that saved her from further harm.

*Message 10* *[Retraction]*. After running a series of tests, doctors have ruled out earlier explanations of the cause of the symptoms. The symptoms were caused by bacterial encephalitis, and the woman is responding well to treatment.

*Message 10* *[Control]*. Ambulance officers who responded commended her friends for their quick response.

*Message 11*. The woman’s father spoke on behalf of the family, stating that they were glad to hear news that she was recovering well, and that they were extremely proud of the strength she had shown.

*Message 12*. The owners of the nightclub have wished the woman well for her recovery and hope she is out of hospital soon.

**Questionnaires**

**Scenario 1—Airplane Landing**

***Memory Questions.***

1. What airport did the airplane land at? (a. Portland; b. Denver; c. Orlando; d. Seattle).
2. Where was the airplane flying to? (a. Washington; b. Los Angeles; c. Boston; d. San Francisco).
3. How many passengers were on board? (a. 530; b. 540; c. 550; d. 560).
4. What type of aircraft was involved? (a. Boeing 747; b. Airbus A380; c. Boeing 777; d. Airbus A319).

***Inference Questions.***

1. Briefly summarise the ‘airplane landing’ article.
2. The pilot’s planning of the flight path should be reviewed. (0-10 scale from “Completely disagree” to “Completely agree”).
3. The accuracy of the pre-flight weather reports should be scrutinized. (0-10 scale from “Completely disagree” to “Completely agree”).
4. The US guidelines for flying in bad weather should be reviewed. (0-10 scale from “Completely disagree” to “Completely agree”).
5. Pilots should receive additional training for flying in bad weather. (0-10 scale from “Completely disagree” to “Completely agree”).
6. “Severe weather forces emergency landing” would be a good headline for this report. (0-10 scale from “Completely disagree” to “Completely agree”).
7. (R) Additional restrictions are not needed when flying in bad weather. (10-0 scale from “Completely disagree” to “Completely agree”).

**Scenario 2—Bushfire**

***Memory Questions.***

1. Where did the bushfire occur? (a. Shepparton, b. Euroa, c. Benalla, d. Kyneton).
2. Why was the fire difficult to control? (a. dense vegetation; b. strong winds; c. dry conditions; d. rough terrain).
3. How many hectares of bushland were burnt? (a. 100,000; b. 25,000; c. 50,000; d. 200,000).
4. What was local resident Casey Hass relieved about? (a. That no one had been harmed; b. That her house had not been affected; c. That her pets had survived; d. That rain had set in).

***Inference Questions.***

1. Briefly summarise the ‘bushfire’ article.
2. Residents are likely to be mistrustful after the bushfire. (0-10 scale from “Completely disagree” to “Completely agree”).
3. It would be lawful for someone to be punished as a result of the bushfire. (0-10 scale from “Completely disagree” to “Completely agree”).
4. The government should spend more resources to prevent arson. (0-10 scale from “Completely disagree” to “Completely agree”).
5. Harsher penalties for arsonists may have prevented the incident. (0-10 scale from “Completely disagree” to “Completely agree”).
6. “Residents on edge after arson causes bushfire” would be a good headline for this report. (0-10 scale from “Completely disagree” to “Completely agree”).
7. (R) No one should be held accountable for the bushfire. (10-0 scale from “Completely disagree” to “Completely agree”).

**Scenario 3—Water Source**

***Memory Questions.***

1. What is the name of the region affected? (a. Harding; b. Shelby; c. Joplin; d. Liberty).
2. What position does Katy Gettys hold? (a. Police officer; b. Mayor; c. Doctor; d. Resident).
3. How many days was intake from the water supply shut down for? (a. 1; b. 5; c. 13; d. 27).
4. What contributed to low water storage levels? (a. drought; b. over-usage; c. containment leak; d. pump failure).

***Inference Questions.***

1. Briefly summarise the ‘water source’ article.
2. The pharmaceutical company should be fined. (0-10 scale from “Completely disagree” to “Completely agree”).
3. Residents who drink from this water supply are likely to have health concerns. (0-10 scale from “Completely disagree” to “Completely agree”).
4. Control measures in riverside industrial areas in Missouri should be tightened. (0-10 scale from “Completely disagree” to “Completely agree”).
5. Residents will be hesitant to drink water from the supply. (0-10 scale from “Completely disagree” to “Completely agree”).
6. “Chemicals pollute drinking water” would be a good headline for this report. (0-10 scale from “Completely disagree” to “Completely agree”).
7. (R) Residents are likely to fully trust the safety of their drinking water. (10-0 scale from “Completely disagree” to “Completely agree”).

**Scenario 4—Nightclub**

***Memory Questions.***

1. What nightclub was the woman partying at? (a. Loft; b. Metro; c. Cable; d. Wire).
2. On average, how many patrons visit the mentioned nightclub on a weekend? (a. 900; b. 1,200; c. 1,500; d. 1,800).
3. What subject did the woman study at the Regent Academy? (a. Photography; b. Interior design; c. Fashion; d. Beauty therapy).
4. Who spoke on behalf of the woman’s family? (a. mother; b. father; c. sister; d. boyfriend).

***Inference Questions.***

1. Briefly summarise the ‘nightclub’ article.
2. It is likely that the incident resulted from an illegal activity occurring at the nightclub. (0-10 scale from “Completely disagree” to “Completely agree”).
3. (R) It is likely that the woman would have needed to go to hospital, even if she hadn’t gone to the nightclub. (10-0 scale from “Completely disagree” to “Completely agree”).
4. The nightclub should introduce a ‘bottled drinks only’ policy. (0-10 scale from “Completely disagree” to “Completely agree”).
5. Someone should be held responsible for the incident. (0-10 scale from “Completely disagree” to “Completely agree”).
6. “Drink spiking incident at popular nightclub” would be a good headline for this report. (0-10 scale from “Completely disagree” to “Completely agree”).
7. (R) Police do not need to investigate the circumstances of the incident. (10-0 scale from “Completely disagree” to “Completely agree”).

**Experiment 2**

**Event Report**

**Event Report—Part 1**

**Welcome to the game.** Welcome to the UWA Memory Lab Detective Game. Are you ready to investigate a crime, Detective? Press the START button below to begin playing.

**Welcome to the crime scene.** You have arrived on the scene of a burglary. Police were called to a home in a middle-class residential neighbourhood after the homeowner, Ms Harter, noticed that her jewellery was missing. The stolen items are reported to include gold chains, gold and silver earrings, rings, and pendants of precious stones including a green sapphire. The initial responding officers identified the living room as the scene of the crime. The scene has been secured and it is ready for your inspection.

**Familiarisation.** As this is your first time as a VR detective, we'll need to familiarise you with the equipment. After clicking the "Next" button below, please look to the left to see another message. Great! Now, press the "Next" button and read the message to your right. Great! Now look in the middle again after clicking the "Next" button.

**How to inspect.** You now need to look around the room for clues. You do not need to move around in order to inspect the clues. In order to inspect a clue, look around for a large magnifying glass icon. When you see it, click on it using the laser pointer to inspect the clue. If you are ready, press "Start" to begin.

**Clue inspection.**

***Clue 1 thread.*** You found a piece of blue thread. The thread may come from a scarf belonging to the homeowner. You are awaiting analysis.

***Clue 2 handprint.*** Partial handprint. It does not match any known offenders in the system.

***Clue 3 broken latch.*** Broken latch. This indicates a likely point of entry for the crime.

***Clue 4 paper.*** Bookmaker's betting slip. Reports from the family indicate the homeowner's son, Evan, has a gambling problem and has accumulated large debts.

***Clue 5 shoeprint.*** Shoeprint, Size 11. Forensics guess it belongs to a male, approximately 6ft. The homeowner's son is 6ft.

***Clue 6 hair.*** A strand of blonde hair. It does not match anyone in the residence.

***Clue 7 cigarette butt.*** Cigarette butt. The homeowner is a non-smoker; however, her son is a known smoker.

***Clue 8 button.*** A red button. This does not match any of the resident's clothing.

**End of inspection no-shift condition.** Thorough work Detective! You have inspected all the clues in the scene. Please wait while we process the case details.

**End of inspection shift condition.** Thorough work Detective! You have inspected all the clues in the scene. Please press the button below to return to HQ to process the case details.

**Event Report—Part 2**

**Welcome back to HQ.** Welcome back to HQ Detective! There's more work to do in the interview room but it is being used right now. They should be done soon—please wait.

**Interview room free.** Great! The interview room is available now. Let's go and complete the paperwork for this case.

**Fill in reports.** There are some reports for you to fill in. Press Start to begin completing the reports.

**Update to case.** Detective, you have been sent some emails with updates on the case. Please click "Start" to view them.

**Retraction emails.**

***Retraction statement 1.*** Analysis from the lab has ruled out the homeowner's scarf as the source of the blue thread.

***Retraction statement 2.*** Examination of an adjacent building's CCTV footage has not revealed anything of use.

***Retraction statement 3.*** Additional information confirmed that one of the jewels stolen was a blue sapphire not a green sapphire as was originally recorded.

***Retraction statement 4.*** Several independent sources confirm that the homeowner's son, Evan, had been out of town on business.

***Retraction statement 5.*** The neighbourhood has been hit with a number of thefts recently. There have been no arrests or leads in these cases so far.

***Retraction statement 6.*** Lab analysis revealed that the hair was not human and belonged to a dog—most likely a golden retriever. The adjacent neighbour reportedly owns a golden retriever.

***Retraction statement 7.*** Police have been unable to locate the jewellery at any local pawn shops***.***

***Retraction statement 8.*** Police are still attempting to determine whether other valuables are missing from the house. The television and a home computer were not disturbed.

**Update completed no-shift condition.** You have finished reading through the updates on the case. We will now head back to HQ to complete the reports.

**Update completed shift condition.** You have finished reading through the updates on the case. Please wait for the interview room to become available.

**Case closed.** Great work, case closed! Your boss will examine your report and look at taking this to the Magistrate's Court. You may remove the headset now. The application will close automatically in a few seconds.

**Memory Questions**

Detective, please answer the following multiple choice questions from memory (press Next to continue).

1. What colour was the button? (a. Blue; b. Green; c. Red; d. Yellow).
2. What colour was the thread? (a. Blue; b. Green; c. Red; d. Yellow).
3. What colour was the hair? (a. Blonde; b. Brunette; c. Black; d. Red).
4. What size was the shoeprint? (a. Size 8; b. Size 9; c. Size 10; d. Size 11).
5. What was broken? (a. A window; b. The door latch; c. A vase; d. The door frame).
6. What colour was the sapphire? (a. Green; b. Blue; c. Pink; d. Orange).

**Inference Questions**

Detective, you will now be asked to make some judgements on a scale from 0 to 10. Try out the slider below to practice the rating task. When you are ready to continue, press Next.

1. How likely are the family to be angry with their son?
2. How likely is the son to feel bad about the incident?
3. How likely are your team to investigate people other than the resident's son? (R)
4. How confident are you that Evan Harter, the resident's son, committed the crime?

**Charge Question**

1. Who will you charge? (a. Evan [son]; b. H Brown [known offender]; c. nobody).
