# Supplementary material for: Examining the role of information integration in the continued influence effect using an event segmentation approach
Source: PLoS One. 2022 Jul 18;17(7):e0271566. doi: 10.1371/journal.pone.0271566 (PMC9292086; doi:10.1371/journal.pone.0271566)
Supplement: Attachment — Submitted filename: Response to Reviewers.docx [file pone.0271566.s002.docx]

**Response to Reviewers**

PONE-D-21-32228

Examining the role of information integration in the continued influence effect using an event segmentation approach

Original comments from action letter are in courier font whereas our replies are in Times Roman.

**Editor**

Thank you for submitting your manuscript to PLOS ONE. I have now received comments on your manuscript from two expert reviewers and I have also studied your manuscript myself. Both reviews were positive, with comments that were constructive and clear. After careful consideration, we feel that it has merit but does not fully meet PLOS ONE’s publication criteria as it currently stands. Therefore, we invite you to submit a revised version of the manuscript that addresses the points raised during the review process.

A couple of points stood out for further comment. I agree with Reviewer 2 that results in the supplementary materials section would be better off in the main results section.

Thank you for inviting us to revise and resubmit our manuscript. We now include the results from the supplementary materials in the main text.

More substantively, like Reviewer 1, I also wondered how the effect used in the power analyses of Experiment 1 was chosen. Furthermore, these f effect sizes can be difficult to understand so I wonder if it can be also explained in more concrete terms (see below, and in relation to the specific test(s) used). I highlight this in particular as in the face of null results an important question to ask is how likely was a type 2 error to have occurred. While the Bayes Factors did give good support for the null hypotheses, the sample sizes used here for a between-subjects design will likely have limited sensitivity to detect small effect sizes. PLOS-ONE is a journal where our policy is to accept null results where the methods are sound, but in these cases I would normally like to see more consideration of statistical power and discussion of the sensitivity of the design to detect an effect if one were present.

Thank you for your comment. The effect size chosen was informed by previous research (e.g., Rinck and Bower [2000] found an effect of *f* = .23). In addition, Brysbaert (2019) recommends *f* = .20 as a default effect size for power analyses in psychological research. We have clarified our power analysis on p. 9, “*The original target sample size was derived using a-priori power analysis [using G*Power 3; 64, also see 65] which suggested that to detect a difference between no-shift and shift conditions inference scores in a t-test, an effect of f = .20 (α = .05; 1-β = .80) would require a sample of 200 participants*.” An advantage of using Bayesian hypothesis testing is that we can find evidence for the null hypothesis, and the inference is conditional on the data observed. The sample size was sufficiently large to support the evidence for the null hypothesis in this experiment.

As I understand it, in the case here I note that the main statistical hypothesis tested in the results of Experiment 1 was a difference in conditions in the size of the inference effect - a main effect in a Bayesian ANOVA, with three conditions. However, the hypotheses in the introduction did not straightforwardly map onto this test, with the following hypotheses:

H1: "both no-shift and shift conditions would reduce reliance on misinformation compared to a no-retraction control"
H2: "integration would be facilitated in the no shift condition... relative to the shift condition"

The exact of test of H1 was unclear to me - would this be the average of the no-shift + shift conditions compared with the control condition? H2 seemed to be more in line with a t-test (as in Experiment 2). I suspect that the study had good power to detect a CIE but may have limited power to detect a modulation of the CIE unless the modulation was large. Relating back to the power analysis used as a justification, it mentions "detecting an effect", but not which one, as there are several on offer here (i.e. the CIE effect and effects associated with the 2 hypotheses). This could be clarified, with more detail on the mapping of your hypotheses to tests. Furthermore, with frequentist tests it is straightforward and informative to conduct a sensitivity analyses of your design - this is not conditional on the effect you observed, but what effect sizes you would have good power to detect given the properties of your design and test used. Given the use of Bayesian analysis this might be less straightforward to conduct, but as mentioned above in the face of a null result greater consideration of sensitivity seems necessary, especially as in the discussion it makes reference to numerical differences in the effects across conditions.

We apologize for the confusion. We have reorganized hypotheses such that the primary hypothesis (previously H2) is now explained first, and the secondary hypothesis (previously H1) explained second.

With respect to the secondary hypothesis (previously H1), it refers to the no-shift and shift conditions inference scores being compared separately to the no-retraction control condition. We have clarified our hypothesis on pp. 8-9, “*A secondary hypothesis was that a retraction (in either shift or no-shift conditions) would reduce reliance on misinformation compared to a no-retraction control, without entirely eliminating its influence, replicating previous work [e.g., 15, 62, 63]*.”

With respect to the analysis regarding the main hypothesis (previously H2), we apologize that this was unclear. In the one-way between-subjects ANOVA we simply compared no-shift and shift conditions inference scores. Although the tests are equivalent, we have now changed this to an independent samples *t*-test to avoid confusion (p. 16): “*A Bayesian independent samples t-test comparing no-shift and shift conditions yielded substantial evidence against a main effect of condition, t(108) = 1.05, BF_01_ = 3.02 (though numerically the no-shift condition had a higher inference score than the shift condition), indicating that the presence (vs. absence) of a spatial event boundary between misinformation and retraction had no measurable impact on participants’ reliance on the misinformation in their inferential reasoning*.”

With respect to the specific effect being tested in the power analysis, this refers to the primary hypothesis (previously H2). We have clarified this on p. 9, “*The original target sample size was derived using a-priori power analysis [using G*Power 3; 64, also see 65] which suggested that to detect a difference between no-shift and shift conditions inference scores in a t-test, an effect of f = .20 (α = .05; 1-β = .80) would require a sample of 200 participants.*”

We have conducted a sensitivity analysis (using G*Power) for an independent samples *t*-test which found that our sample was large enough to detect an effect size of *f* = .27. Additionally, in Experiment 1 the numeric effect between no-shift and shift conditions was in the opposite direction to our hypothesis, which speaks against a false-negative.

Finally, we have conducted a sensitivity analysis for the Bayesian statistics. Conditioning on states of the world is not philosophically consistent with the Bayesian approach, but it is useful to show that the data we have are able to pull apart H0 and H1. We simulated 1000 datasets of the same size as our data from either H0 (*f* = 0) or H1 (*f* = 0.2) and obtained a Bayes Factor for each data set. The figure below shows a receiver operating characteristic (ROC) function looking at the mass of the resulting Bayes Factor distributions below the curve for differing possible BF criteria (this is also philosophically inconsistent with the Bayesian approach, and is used similar to indicate how much the mass of one curve is above the other). The figure shows that the test is relatively sensitive, in that with the amount of data we have we would expect substantially different Bayes Factors under H0 vs H1.

**Reviewer 1**

The topic of the mechanisms underlying the negative proactive effects of misinformation on memory for correct information is quite timely given the high prevalence of misinformation in everyday life. The present study takes a unique approach to illuminating the role of various encoding and retrieval mechanisms in the updating of memories for inaccurate information. I appreciated the creative connection between event segmentation theory and the continued influence effect as well as the innovative experimental procedure. However, the study had some key limitations, such as the ambiguity in results across experiments. On balance, this package seems appropriate for the journal with the caveat that the inconsistency in results across experiments should be highlighted as an impediment for clear interpretation. Below, I describe some concerns and offer specific comments that I hope will help improve the manuscript.

Thank you for your feedback. We have addressed these points in our responses to your comments below.


One general issue was that the Introduction did not summary a relevant literature on the role of retrieval during encoding in information integration. The present study appears to examine how disruptions in the contact between misinformation and subsequent retractions impacts memory updating. There are studies from the memory and discourse comprehension literatures that should inspire predictions in the current experiments. Specifically, there is recent memory work from Wahlheim and Zacks (2019), Wahlheim et al. (2020), and Cohn-Sheehy et al. (2021) on this topic. There is also earlier discourse comprehension work from Kintsch and more recent work from Kendeou and colleagues that may be relevant here. The authors may consider how the notion of integrative encoding (information integration) in those studies fits in here. In my view, the findings from these studies showing that memory updating is more effective when recent information elicits retrieval of earlier information also leads to the prediction that reducing the accessibility of earlier information via event boundaries should lead to fewer retrievals and therefore less integrative encoding. This perspective could provide a more complete mechanistic motivation for the hypotheses given in the present study.

Thank you for your suggestion. This is exactly the point we were trying to make, based on Kendeou’s model (her work was cited as references 21 and 22). We now realize that our descriptions were too terse. We have made the rationale behind the prediction clearer by adding an explanation in the new “Current Study” section on pp. 7-8: (“*According to the mental-model account and event segmentation theory, a spatial event boundary between the encoding of the misinformation and retraction should disrupt retraction integration and model updating. To illustrate, the work of Kendeou [21, 22, also see 59, 60, 61] has* *shown that memory updating is facilitated when the initial misinformation and its subsequent correction are co-activated in memory and a conflict detected. This should be made less likely by an event boundary occurring between the two pieces of information, as encountering the correction may fail to trigger successful retrieval of the earlier misinformation. In turn, this could hinder integration and mental-model revision, thus resulting in increased misinformation reliance.*”). We agree that recent Wahlheim & Zacks, Wahlheim et al. and Cohn-Sheehy et al. papers provide additional evidence for the importance of integration, and have therefore added references to those papers as well.

Specific Comments:

p. 5, line 99: Do event boundaries decrease the “availability” or “accessibility” of information in memory?

We now use the term “accessibility” throughout the paper.

p. 7: It would be great if there was a section here that synthesized the literature review and provided an overarching description of “The Present Study” to give the reader a better understanding of why the three literatures in the Introduction were considered.

Thank you for the suggestion. We now include this on pp. 7-8, “*The current study investigated the effect of spatial event segmentation boundaries on information integration and updating in a CIE paradigm.* *According to the mental-model account and event segmentation theory, a spatial event boundary between the encoding of the misinformation and retraction should disrupt retraction integration and model updating. To illustrate, the work of Kendeou [21, 22, also see 59, 60, 61] has* *shown that memory updating is facilitated when the initial misinformation and its subsequent correction are co-activated in memory and a conflict detected. This should be made less likely by an event boundary occurring between the two pieces of information, as encountering the correction may fail to trigger successful retrieval of the earlier misinformation. In turn, this could hinder integration and mental-model revision, thus resulting in increased misinformation reliance. Across two experiments, misinformation and retractions were presented with or without a spatial event boundary between their encoding. To employ the spatial event boundary, participants either moved physically (Experiment 1) or virtually (Experiment 2) between spatial contexts. … Both experiments included inference questions to assess misinformation reliance*.”

pp. 7 and 8, lines 147-152: What was the rationale for using a between-subjects design instead of a within-subjects design? Was there any concern about sampling from multiple populations for the experimental (undergraduates) and control (MTurk workers) conditions? More generally, I could not understand from the description of the experiment what the control condition was intended to assess with respect to the main hypotheses.

We carefully considered both within-subjects and between-subjects designs in designing this research. A within-subjects design was our preferred default in being more powerful. However, a substantial concern for us with the within-subjects design was that the event reports for each condition would always share a context (e.g., participants would read the entire no-shift event report and part 1 of the shift event report in Room A and part 2 of the shift event report in Room B). Ultimately, we decided that a between-subjects design would allow for a cleaner manipulation.

With respect to sampling from different populations, this was taken into consideration; however, the main purpose of the experiment was to contrast the two experimental conditions, and we did not conduct any analysis involving the control condition, which was included as a check on the effectiveness of the retraction (as indicated on p. 16).

p. 8, Participants: How did the authors choose the effect size that was targeted in the power analysis?

The effect size chosen was informed by previous research (e.g., Rinck and Bower [2000] found an effect of *f* = .23). In addition, Brysbaert (2019) recommends *f* = .20 as a default effect size for power analyses in psychological research.

p. 9, Event reports: It would be great to have more information about what motivated the structure of the retractions. Specifically, the example here does not include details from the original misinformation statement. Was this done purposefully to decrease the contact between retractions and misinformation to increase the chances that the event boundary manipulation would reduce the accessibility of misinformation? I have a similar query about the materials in Experiment 2.

Thank you for your question. The event reports were based on those from Ecker et al. (2017) who compared the effectiveness of retractions which explicitly referred to misinformation to retractions that avoided repeating the misinformation. Ecker et al. found that explicit retractions were more effective in reducing misinformation reliance, presumably by facilitating conflict detection and model updating. As such, as you interpreted correctly, we decided to not explicitly refer to the misinformation within the retraction as this may facilitate integration, which would likely have reduced the effect of the event boundary manipulation. We now explain this on p. 10, “*It was decided to not explicitly refer to the misinformation within the retraction as previous research [62] has found that explicit retractions were more effective in reducing misinformation reliance, presumably by facilitating conflict detection and model updating. As such, referring explicitly back to the misinformation may facilitate integration, which may reduce the impact of the event boundary manipulation*.”

p. 10, lines 196-201: Do the authors worry that providing response alternatives on the questionnaire had reactive effects on subsequent memory for event details? I have a similar query about the materials in Experiment 2.

We were not overly concerned as the memory questions assessed memory for irrelevant/filler details that were not central to the misinformation and retraction. We now include this as a potential limitation on p. 18, “*In addition, it might be speculated that the inclusion of response alternatives in the memory questions may have facilitated general recall of the event, thereby indirectly reducing the impact of the boundary manipulation on inferential reasoning*.”

p. 10, line 220: I believe the monitor was a 21.5-inch (not 31.5 inch).

Thank you! We have changed the monitor dimensions.

p. 11, Procedure: What motivated the decision to administer the final memory test in a unique room (i.e., Room C)? I had the impression that the effects of spatial shift would be most pronounced if the final testing room context matched the retraction encoding context.

As discussed on p. 7, the effect of spatial event boundaries does not appear to be simply due to context-dependent memory as Radvansky and colleagues found that memory was affected even when individuals were tested in the original room of encoding. However, we still decided to administer the final test in a unique room to keep testing conditions constant while allowing for room order counterbalancing.

p. 12, Memory scores: Are the authors concerned that providing response alternatives on a multiple-choice memory test rescues impaired event details accessibility post-boundary? In other words, could available but less accessible details be made more accessible with the presentation of copy cues during the test?

We assume that this is essentially the same point as the one made previously about the multiple-choice questions. As mentioned above, we were not overly concerned as the memory questions assessed memory for irrelevant/filler details that were not central to the misinformation and retraction, and were included to ensure general engagement with the task. We acknowledge this as potential limitation on p. 18, “*In addition, it might be speculated that the inclusion of response alternatives in the memory questions may have facilitated general recall of the event, thereby indirectly reducing the impact of the boundary manipulation on inferential reasoning*.”

p. 13, lines 287-290: I was uncertain how the comparison presented here provided support for a continued influence effect. Shouldn’t that require a comparison of misinformation correction items with control items for which no misinformation had appeared? If I have just missed something, it would be great if the authors could explain that here.

While one-sample *t*-tests against zero provide some support for a CIE, we acknowledge that zero may not be an appropriate baseline when using rating scales, and we cannot make strong claims about the presence of a continued influence effect without a no-misinformation condition. We acknowledge this on p. 16, “*We acknowledge that zero may not be an appropriate baseline when using rating scales, but use zero in the absence of any other obvious baseline*.” We have also softened our conclusion in the general discussion and now include this as a suggestion for future research on p. 28, “*However, while retractions appeared effective, both studies found that they did not completely eliminate reliance on misinformation, providing some evidence for a CIE, again in line with previous research [2, 6, 14, 20, 89]. Future research could include a no-misinformation condition in order to formally establish the presence of a CIE*.”

p. 14, Discussion: Echoing a concern above, I am not confident that the comparison of experimental groups from one population should be compared directly with a control group from another population. Also, for the possible explanations as to why Experiment 1 failed, the authors may consider my point above that a recognition task could rescue what would otherwise be inaccessible memories of event details following spatial shifts. The authors allude to this possibility in lines 328-329.

We did not conduct an explicit comparison of the no-shift and shift conditions with the control condition for the reason stated. We simply did a rough baseline comparison as a manipulation check, in line with a-priori intentions, as stated on p. 16.

As stated above, we now include the possibility that the memory questions may have increased accessibility of the event as a limitation on p. 18.

pp. 21 and 22: I was confused about the conversion of charge scores into 0 and 10 values. Could the authors have treated this as a 1/0 variable and used some type of logistic regression approach?

The charge scores were converted into 0 and 10 values to maintain consistent scaling with the rating scale inference items. We have clarified this on p. 24, “*Charge scores were based on the multiple-choice charge question. Responses were converted into a score of 0 or 10 (to maintain consistent scaling with the rating scale inference items): ….*” With respect to our analysis choice, in our opinion a chi-square analysis is an appropriate test regardless of scale, and we do not see any benefit to using a logistic regression.

General Discussion: This section is a bit shorter than I am accustomed to reading in multi-experiment reports. The authors might consider connecting their findings to the literature more broadly here.

Thank you for the suggestion. We have extended our discussion on p. 29, “*Together, these studies have implications for event segmentation theory and the replicability and generalizability of event segmentation effects to a CIE context. In particular, the findings show that event segmentation may only impact specific retrieval processes but not downstream processes such as integration and updating. Future research may wish to investigate the effect of spatial event boundaries within the event reports (e.g., a protagonist moving locations), as opposed to being applied externally to the participant [41, 77]. Future* *research could also use other types of event boundaries (e.g., shifts in temporal context) [92].*”

**Reviewer 2**

In two behavioral studies, Sanderson and colleagues utilized an event segmentation approach to investigate the persistence of misinformation. Given the robustness of the continued influence effect, finding new ways to investigate the phenomenon is a very worthwhile pursuit. Overall, I find the procedure and results easy to follow, and I think the VR detective framing (Experiment 2) is quite clever. Below are a few comments and suggestions which may help further strengthen the paper.

Thank you for your feedback. We have addressed each of your comments below.

Introduction:
1. As I progressed through the introduction, I was anticipating a study that manipulated event segmentation within the context of the narratives. As such, I was surprised when I came to find out that the manipulation was segmentation of the encoding environment. Further motivation and clarification of that manipulation choice in the introduction will be important.

Thank you for the suggestion. We have clarified our decision of segmenting the encoding environment rather than narrative context manipulations on pp. 7-8, “*Across two experiments, misinformation and retractions were presented with or without a spatial event boundary between their encoding. To employ the spatial event boundary, participants either moved physically (Experiment 1) or virtually (Experiment 2) between spatial contexts. Physical/virtual spatial context changes were chosen so as to accommodate the event report format typically used in CIE studies, which do not lend themselves to narrative spatial shifts. For example, in contrast to the story-based narratives typically used in event segmentation studies, event reports are by nature less linear in terms of structure and less confined to a particular context/person (e.g., written in third person with input from multiple sources), which makes it difficult to implement event boundaries*.”

I should add that although I am generally familiar with the event segmentation literature, I was unfamiliar with the “location updating effect” described on page 6. When I read the description of “…when an agent moves from one location to another…”, I was assuming that the “agent” refers to the protagonist of the narrative. Clarifying that section -- and further explaining how this goes beyond the traditional context-dependent memory effects -- will go a long way towards minimizing potential confusion.

We have clarified this section on p. 6, “*In a series of experiments conducted by Radvansky and colleagues [39, 51, 52], participants navigated a series of rooms while picking up and setting down different objects in each room. During this, participants’ recognition memory for the objects was tested. The critical factor of interest was whether or not participants shifted to a new room between encoding and test. These studies found that memory for objects was less accurate and slower following a shift in location compared to when there was no shift. In other words, the accessibility of information from the previous room decreased after a spatial event boundary had been crossed. This finding was even more pronounced when participants moved through multiple rooms, therefore crossing multiple event boundaries [51].*”

We have expanded our discussion of context-dependent memory on p. 7, “*Furthermore, the effect does not appear to be simply due to context-dependent memory which would suggest that returning to the location where information was initially learned would improve recall [e.g., 57, 58], as memory was affected even when individuals were tested in the original room of encoding [51].*”

Experiment 1:
2. In coding the open-ended inference question, the authors opted for a broad definition of “no misinformation reliance”, page 12: “If participants recalled the retraction and/or the alternative, they received a score of 0 (indicating no misinformation reliance) irrespective of whether they also recalled the critical information…”

Recent work has shown that there may be an important distinction between different operationalizations of “continued influence” ([https://doi.org/10.1186/s41235-021-00335-9](https://aus01.safelinks.protection.outlook.com/?url=https%3A%2F%2Fdoi.org%2F10.1186%2Fs41235-021-00335-9&data=04%7C01%7Cjasmyne.sanderson%40research.uwa.edu.au%7C996dc3d9025649af435d08d9fd02e5a3%7C05894af0cb2846d8871674cdb46e2226%7C0%7C0%7C637819011156560579%7CUnknown%7CTWFpbGZsb3d8eyJWIjoiMC4wLjAwMDAiLCJQIjoiV2luMzIiLCJBTiI6Ik1haWwiLCJXVCI6Mn0%3D%7C3000&sdata=vrzct4CaJzQw5JctvxAfM44UgcGFVuXVwdqDAjhaInY%3D&reserved=0)). If the open-ended responses were coded differently -- for example, 0 when they recalled only the retraction and/or alternative; 5 points when they recalled retraction and/or alternative AND critical information; 10 = critical information only -- will the patterns look different? This may be a more nuanced way to distinguish among the varying levels of success at integration. This maybe an interesting exploratory analysis, especially since the initial analyses did not yield any effect of segmentation conditions.

Thank you for the suggestion. We have recoded the open-ended inference item as per your suggestion and the results of an independent sample *t*-test with the combined open-ended and rating scale inference items are very similar to our original analysis (*t*[108] = 1.05, *BF*_01_ = 3.03). When the analysis is performed with just the open-ended inference item, the outcome is ambiguous with *t*(108) = 2.01, *BF*_01_ = 0.83 (original coding) and *t*(108) = 1.73, *BF*_01_ = 1.30 (recoded). We have added a reference to the Kan et al. paper.

3. Minor point: I assume the researchers converted the open-ended question score to 0 or 10 so that the scale would match the close-ended questions? It would be helpful to include an explicit statement about that in the scoring section.

Yes, we have clarified this on p. 14, “*The coded scores were then converted into an inference score of 0 or 10 to maintain the same scaling as the rating scale inference questions…*”.

Experiment 2:
4. In Part 2 of the report, retractions of non-critical information were introduced alongside retractions of critical information. Please clarify the rationale for and utility of this design choice.

We included retractions of non-critical information in order to not overly highlight the retractions of the critical information, as well as to stay consistent with the typical written event reports used in CIE studies which present retractions alongside other filler information. We have clarified this on p. 21, “*Retractions of non-critical information were included in order to not overly highlight the retraction of the critical information, and to maintain consistency with previous CIE research, which tends to present retractions not in isolation but in the context of other filler information [e.g., 80, 81]*.”

5. Unless dictated by a prescribed word limit, I strongly encourage the authors to incorporate the Results reported in the Supplement into the main text.

We now include the results from the supplementary materials in the main text.

General Discussion:
6. On page 24, the authors wrote, “Both experiments found that a retraction was effective in reducing reliance on misinformation …” Since Experiment 2 did not include a baseline (no retraction) condition, this claim does not apply to Experiment 2. Please rephrase.

We have rephrased this statement to: “*The results of Experiment 1 suggested that a retraction was effective in reducing reliance on misinformation, as reflected in participants’ inferential reasoning scores, consistent with previous research [1, 5, 15, 62, 63]*.” (p. 28).

7. The paper will benefit from some discussion of the future utility of using the event segmentation approach as a way to understand CIE.

We now include a discussion and suggestions for future research about using an event segmentation approach to understand the CIE on p. 29, “*Together, these studies have implications for event segmentation theory and the replicability and generalizability of event segmentation effects to a CIE context. In particular, the findings show that event segmentation may only impact specific retrieval processes but not downstream processes such as integration and updating. Future research may wish to investigate the effect of spatial event boundaries within the event reports (e.g., a protagonist moving locations), as opposed to being applied externally to the participant [41, 77]. Future research could also use other types of event boundaries (e.g., shifts in temporal context) [92].*”

References

[1] Chan MPS, Jones CR, Hall Jamieson K, Albarracín D. Debunking: A meta-analysis of the psychological efficacy of messages countering misinformation. Psychol Sci. 2017;28:1531-46. <https://doi.org/10.1177/0956797617714579>

[2] Johnson HM, Seifert CM. Sources of the continued influence effect: When misinformation in memory affects later inferences. J Exp Psychol Learn Mem Cogn. 1994;20(6):1420-36. <http://doi.org/10.1037/0278-7393.20.6.1420>

[5] Walter N, Murphy ST. How to unring the bell: A meta-analytic approach to correction of misinformation. Commun Monogr. 2018:1-19. <https://doi.org/10.1080/03637751.2018.1467564>

[6] Walter N, Tukachinsky R. A meta-analytic examination of the continued influence of misinformation in the face of correction: how powerful is it, why does it happen, and how to stop it? Commun Res. 2020;47:155-77. <https://doi.org/10.1177/0093650219854600>

[14] Ecker UKH, Lewandowsky S, Tang DTW. Explicit warnings reduce but do not eliminate the continued influence of misinformation. Mem Cognit. 2010;38(8):1087-100. <http://doi.org/10.3758/mc.38.8.1087>

[15] Swire B, Ecker UKH, Lewandowsky S. The role of familiarity in correcting inaccurate information. J Exp Psychol Learn Mem Cogn. 2017;43:1948-61. <https://doi.org/10.1037/xlm0000422>

[20] Ecker UKH, Lewandowsky S, Swire B, Chang D. Correcting false information in memory: Manipulating the strength of misinformation encoding and its retraction. Psychon Bull Rev. 2011;18(3):570-8. <https://doi.org/10.3758/s13423-011-0065-1>

[21] Kendeou P, Butterfuss R, Kim J, Van Boekel M. Knowledge revision through the lenses of the three-pronged approach. Mem Cognit. 2019;47:33-46. <https://doi.org/10.3758/s13421-018-0848-y>

[22] Kendeou P, Walsh EK, Smith ER, O'Brien EJ. Knowledge revision processes in refutation texts. Discourse Process. 2014;51:374-97. <http://doi.org/10.1080/0163853X.2014.913961>

[39] Radvansky GA, Copeland DE. Walking through doorways causes forgetting: Situation models and experienced space. Mem Cognit. 2006;34(5):1150-6. <https://doi.org/10.3758/BF03193261>

[41] Rinck M, Bower GH. Temporal and Spatial Distance in Situation Models. Mem Cognit. 2000;28(8):1310-20. <https://doi.org/10.3758/BF03211832>

[51] Radvansky GA, Krawietz SA, Tamplin AK. Walking through doorways causes forgetting: Further explorations. Q J Exp Psychol. 2011;64(8):1632-45. <https://doi.org/10.1080/17470218.2011.571267>

[52] Radvansky GA, Tamplin AK, Krawietz SA. Walking through doorways causes forgetting: Environmental integration. Psychon Bull Rev. 2010;17(6):900-4. <https://doi.org/10.3758/PBR.17.6.900>

[57] Godden DR, Baddeley AD. Context‐dependent memory in two natural environments: On land and underwater. Br J Psychol. 1975;66(3):325-31. <https://doi.org/10.1111/j.2044-8295.1975.tb01468.x>

[58] Thomson DM, Tulving E. Associative encoding and retrieval: Weak and strong cues. J Exp Psychol. 1970;86(2):255-62. <https://doi.org/10.1037/h0029997>

[59] Wahlheim CN, Alexander TT, Peske CD. Reminders of Everyday Misinformation Statements Can Enhance Memory for and Beliefs in Corrections of Those Statements in the Short Term. Psychol Sci. 2020;31(10):1325-39.

[60] Wahlheim CN, Zacks JM. Memory Guides the Processing of Event Changes for Older and Younger Adults. J Exp Psychol Gen. 2019;148(1):30-50.

[61] Cohn-Sheehy BI, Delarazan AI, Reagh ZM, Kim K, Barnett AJ, Zacks JM, et al. The hippocampus constructs narrative memories across distant events. Current Biology. 2021;31:4935-45.

[62] Ecker UKH, Hogan JL, Lewandowsky S. Reminders and repetition of misinformation: Helping or hindering its retraction? J Appl Res Mem Cogn. 2017:1-8. <http://doi.org/10.1016/j.jarmac.2017.01.014>

[63] Ecker UKH, O'Reilly Z, Reid JS, Chang EP. The effectiveness of short‐format refutational fact‐checks. Br J Psychol. 2020;111(1):36-54. <https://doi.org/10.1111/bjop.12383>

[64] Faul F, Erdfelder E, Lang AG, Buchner A. G* Power 3: A flexible statistical power analysis program for the social, behavioural, and biomedical sciences. Behav Res Methods. 2007;39(2):175-91. <https://doi.org/10.3758/BF03193146>

[65] Brysbaert M. How Many Participants Do We Have to Include in Properly Powered Experiments? A Tutorial of Power Analysis with Reference Tables. J Cogn. 2019;2(1):1-38. <https://doi.org/10.5334/joc.72>

[77] Zwaan RA, van Oostendorp H. Do readers construct spatial representations in naturalistic story comprehension? Discourse Process. 1993;16(1-2):125-43. <https://doi.org/10.1080/01638539309544832>

[80] Ecker UKH, Rodricks AE. Do False Allegations Persist? Retracted Misinformation Does Not Continue to Influence Explicit Person Impressions. J Appl Res Mem Cogn. 2020;9(4):587-601.

[81] Sanderson JA, Gignac GE, Ecker UKH. Working memory capacity, removal efficiency and event specific memory as predictors of misinformation reliance. J Cogn Psychol. 2021;33(5):518-32. <https://doi.org/10.1080/20445911.2021.1931243>

[92] Sanderson JA, Farrell S, Ecker UKH. Examining the Reproducibility of Temporal Event Boundaries in Continued Influence Effect and Event Segmentation Paradigms. Psyarxiv [preprint] 2022:1-84. Available from: <https://psyarxiv.com/6zn2a>
