# Supplementary material for: Examining the role of information integration in the continued influence effect using an event segmentation approach
Source: PLoS One. 2022 Jul 18;17(7):e0271566. doi: 10.1371/journal.pone.0271566 (PMC9292086; doi:10.1371/journal.pone.0271566)
Supplement: Attachment — Submitted filename: Response to Reviewers.docx [file pone.0271566.s003.docx]

**Response to Reviewers**

PONE-D-21-32228R1

Examining the role of information integration in the continued influence effect using an event segmentation approach

Original comments from action letter are in courier font whereas our replies are in Times Roman.

**Editor**

Thank you for submitting your manuscript to PLOS ONE. After careful consideration, we feel that it has merit but does not fully meet PLOS ONE’s publication criteria as it currently stands. Therefore, we invite you to submit a revised version of the manuscript that addresses the points raised during the review process.

I was not able to secure the two previous reviewers to review the re-submission. To expedite the review process, I have considered the revised submission and your responses, and have the following comments below. These are focused on analyses and corresponding conclusions in response to revisions or outstanding issues from the original submission.

Thank you for your comments and the opportunity to revise our manuscript. We have addressed each of your points below.

1. Myself and Reviewer 1 asked for a justification for the sample size. This was provided in the cover letter but not in the revisions. Could the rationale be provided in the manuscript. If mentioning a previous finding as a justification, could you provide more detail (e.g., exactly which result/test in the Rinck and Bower paper is being used as justification).

Thank you for your suggestion. We now include the rationale within the manuscript on p. 9, “The original target sample size was derived using a-priori power analysis [using G*Power 3; 64, also see 65] which suggested that to detect a difference between no-shift and shift conditions inference scores in a *t*-test, an effect of *f* = .20 (α = .05; 1-β = .80) would require a sample of 200 participants. The effect size of *f* = .20 was chosen based on Brysbaert [65], who recommends *f* = .20 as a default effect size for power analyses in psychological research. In addition, previous research [41] found an effect of *f* = .23 when comparing no-boundary and boundary conditions using an ANOVA.”

2. Further in regard to the justification, this appears based on a frequentist power analyses with a p < .05 decision threshold, which does not match the inference procedure and tests used in the paper. Could the rationale of this approach be described in the paper.

We initially planned to use frequentist tests for the analyses, hence the frequentist power justification. However, during data collection we decided to “peek” at the data after obtaining half of it and committed to use Bayesian statistics due to this. This is explained on p. 9: “Due to difficulties in recruiting participants, it was recognised that a sequential Bayesian approach would be appropriate to balance the resource-intensive nature of testing against the evidence gained. Accordingly, the data were subjected to Bayesian sequential testing after testing half the number of planned participants, and committing to Bayesian statistics for inference beyond that point [66, 67].”

3. "All Bayesian analyses were conducted in JASP, using default priors.". There was previously some text which described the priors but this was removed. A reproducible analysis script is not available and the JASP version was not given. The default priors in JASP could be subject to change, and hence reproducibility of the analyses would be compromised. Therefore could some information on the priors be provided in the manuscript.

We have re-added the text describing the JASP default priors on p. 14, “All Bayesian analyses were conducted in JASP, using default priors (fixed-effects scale parameter *r* = 0.5; random-effects scale parameter *r* = 1).” and p. 16 “A Bayesian independent samples *t*-test (with default priors: fixed-effects scale parameter *r* = 0.5; random-effects scale parameter *r* = 1)…”.

4. "A Bayesian one-way between-subjects ANOVA confirmed there were no differences between no-shift and shift conditions, F(1, 108) = .02, BF01 = 4.91." The word significant was removed in revisions. I understand the reason for this change since a NHST test was not performed. However, there was a numerical difference so this statement is incorrect. Similar comments apply to the results on p. 25 and perhaps elsewhere, e.g., "a Bayesian independent-samples t-test confirmed there was no difference between conditions, t(98) = .28, BF01 = 4.58". The description of the analysis on p.14 describes ranges of Bayes Factors for which qualitative labels for evidence are assigned based on conventions established in the literature. However, the inferential process that leads to the conclusion of "no difference" based on a BF value is not described, and the pre-registration does not articulate this either. In frequentist analysis the phrase "statistically significant difference" is used to qualify such statements, but such terminology to indicate qualification appropriate to Bayesian hypothesis testing is (mostly) absent here. Throughout, could results be described in a way that it is more clear as to what inference is being performed (with explanation of the inference procedure earlier on if necessary), and to qualify statements the results, i.e., it would seem more appropriate to talk about evidence for effects, or more how the Bayes Factor favours one model over another, rather than categorical statements of a presence/absence of an effect.

Thank you for your suggestion. We now incorporate the Bayesian descriptors more consistently into our results.

5. I apologise but I am still confused on the use of the ANOVA. On page 15 where this ANOVA is described, the means for three conditions were provided (i.e. including the control), but the following statement of a description of just a difference between two conditions (no-shift and shift), which would result from an ANOVA with two levels or a t-test. The following analysis on page 16 again described means for the control conditions along with shift and no-shift conditions, but here t-tests were used. My confusion here is that it does not appear that the control condition was included in any statistical analyses, and therefore I was unclear on the purpose of having it. Could this be explained in the text.

To avoid confusion, we have changed this analysis to an (equivalent) *t*-test, “A Bayesian independent samples *t*-test provided substantial evidence against a difference between no-shift and shift conditions, *t*(108) = -.14, *BF_01_* = 4.91.” (p. 15).

We originally conducted an ANOVA comparing the control condition inference scores with the inferences scores from the no-shift and shift conditions; however, after further thinking we decided to omit this analysis because the control condition came from a separate and non-matched online sample. As such the control condition was included as a simple numerical check on the effectiveness of the retraction (p. 16), “Numerically, both no-shift and shift conditions had lower inference scores than the no-retraction control, suggesting some effect of the retraction.”

6. Page 26 reports Bayesian chi-square tests. There is not sufficient detail to allow reproducibility of these analyses in the manuscript. Could more explanation be provided, including priors used.

We now provide additional information on p. 26, “To test the hypothesis that the no-shift condition would have lower misinformation reliance than the shift condition, a Bayesian chi-square test (using the default prior: *a* = 1) …”

7. I did wonder why the approach taken in Experiment 2 was not used in Experiment 1 - an effect was found in the opposite direction in both cases but only Experiment 2 was a one-tailed test performed. I presume that the reason was that that in Experiment 2 there was weak evidence for the alternative hypothesis, and moderate evidence for the null in Experiment 1. But I wondered how the results of Experiment 1 would change (i.e. make the null findings more equivocal). I wonder if this was considered, and if it might be helpful, but I am not suggesting this is necessary.

Thank you for your suggestion. We now report one-tailed tests for both Experiment 1 and Experiment 2 to better align analysis with our hypothesis and for the sake of consistency. The sentence on p. 17 now reads: “In addition, a one-tailed test (specifying H_1_ as NS < S) was run to quantify the evidence against the directional hypothesis. This provided strong evidence against H_1_, *t*(108) = 1.05, *BF*_01_ = 9.39.”

8. **Sensitivity to small effect sizes:**

- Previously I asked for more consideration of design to detect small effect sizes. The response letter stated:
- "We have conducted a sensitivity analysis (using G*Power) for an independent samples t-test which found that our sample was large enough to detect an effect size of f = .27."
- The purpose of a sensitivity analysis would be to see the range of possible effect sizes that your design had sensitivity to detect. That you had a good power (in frequentist terms) to detect medium to large effects (i.e. f > .2) was not the issue being raised. A further sensitivity analysis was provided in the response editor letter compares H0 with f = 0 with H1 with f = 2 (d = .4). This is comparing a null effect with a medium-ish effect. The point I wanted to see addressed or discussed was the sensitivity of your design to smaller effects.
- "Additionally, in Experiment 1 the numeric effect between no-shift and shift conditions was in the opposite direction to our hypothesis, which speaks against a false-negative."
- Three possibilities can be considered to exist here. There is a true effect in the opposite direction to that predicted, a true null effect, or a true effect in the predicted direction. While it would be unlikely to find a Type S error (i.e., an error of the wrong sign) with a large effect in the predicted direction, the probability of this occurring with a smaller effect is non-negligible. The balance of evidence may favour one (or two) of these accounts over the other, but there does not seem to be very strong evidence to rule out any of these possibilities.
- Note, in Experiment 1, the difference in conditions appeared to be around d = .2, based on the difference in the means and an approximate calculation of the SD. Like Experiment 2, the effect was in the opposite direction as predicted. The BF01 was 3.02. A Bayes Factor provides a continuous measure, but this value is very close to the 3 threshold by some conventions the evidence could be described as anecdotal. Although numerical differences are highlighted in the text, the conclusions sometimes steer towards a more categorical interpretation, e.g. wordings such as "no effect, e.g. " no measurable impact", "failed to find an effect". Yet, with a frequentist analysis, the power would be approximately .18 for a similar sized effect (i.e., in r power.t.test(n=54, d=.2, type="two.sample"). The actual power is unknown, but my point is that a Bayesian analysis cannot overcome the same issue that a frequentist analysis has with detecting small effects with a limited sample size - in Bayesian terms, the probability of finding convincing evidence or misleading evidence (based on some threshold of evidence, e.g.  [Schönbrodt](https://aus01.safelinks.protection.outlook.com/?url=https%3A%2F%2Flink.springer.com%2Farticle%2F10.3758%2Fs13423-017-1230-y%3F%2520utm_campaign%3D8_ago1936_psbr%2520vsi%2520art08%26%2520utm_content%3D2062018%2520%26%2520utm_medium%3Dother%2520%26%2520utm_source%3Dother%2520%26wt_mc%3DOther.Other.8.CON1172.PSBR%2520VSI%2520Art08%2520%26error%3Dcookies_not_supported%26code%3Dde83092d-bf8c-4b90-afbf-717da7041d82%23auth-Felix_D_-Sch_nbrodt&data=05%7C01%7Cjasmyne.sanderson%40research.uwa.edu.au%7C966b80ecdcc44de86a6908da399e5aaa%7C05894af0cb2846d8871674cdb46e2226%7C0%7C0%7C637885649540085717%7CUnknown%7CTWFpbGZsb3d8eyJWIjoiMC4wLjAwMDAiLCJQIjoiV2luMzIiLCJBTiI6Ik1haWwiLCJXVCI6Mn0%3D%7C3000%7C%7C%7C&sdata=tlEnqGMQfUUOGorNwd17yjoRpoFScxH%2FWHxMQfwxplA%3D&reserved=0) & [Wagenmakers](https://aus01.safelinks.protection.outlook.com/?url=https%3A%2F%2Flink.springer.com%2Farticle%2F10.3758%2Fs13423-017-1230-y%3F%2520utm_campaign%3D8_ago1936_psbr%2520vsi%2520art08%26%2520utm_content%3D2062018%2520%26%2520utm_medium%3Dother%2520%26%2520utm_source%3Dother%2520%26wt_mc%3DOther.Other.8.CON1172.PSBR%2520VSI%2520Art08%2520%26error%3Dcookies_not_supported%26code%3Dde83092d-bf8c-4b90-afbf-717da7041d82%23auth-Eric_Jan-Wagenmakers&data=05%7C01%7Cjasmyne.sanderson%40research.uwa.edu.au%7C966b80ecdcc44de86a6908da399e5aaa%7C05894af0cb2846d8871674cdb46e2226%7C0%7C0%7C637885649540085717%7CUnknown%7CTWFpbGZsb3d8eyJWIjoiMC4wLjAwMDAiLCJQIjoiV2luMzIiLCJBTiI6Ik1haWwiLCJXVCI6Mn0%3D%7C3000%7C%7C%7C&sdata=9ouEq5jskQ3Q%2BcYUQjRoOtym1ywRMtuA68txdsBCJTs%3D&reserved=0), 2018).
- It seems to me that the evidence seems pretty convincing that there is not a medium or large effect, but the evidence for ruling out a small effect is not convincing. And based on the results of Experiment 2, a small effect in the opposite direction as predicted in Experiment 1 does seem possible. I do note that that in terms of your conclusions, either a null effect or an effect in the opposite direction in Experiment 1 does not change your substantive conclusions, hence this may be considered a minor point, and it is quite a generic one - i.e. most psychology designs use sample sizes in a range that make it difficult to detect small effects. Consequently, I don't think a sensitivity analysis is necessary in a revision, but this point can easily be addressed by more judicious use of language to describe evidence for effects in the results (as mentioned in comments above) and corresponding language to match the description of results in the discussion, and what can and cannot be plausibly ruled out.

Thank you for your comment. As mentioned above, we have incorporated Bayesian descriptors more consistently into our results. We have also rephrased our conclusions in relation to the results. In relation to the specific examples provided by the editor, we now report: “A Bayesian independent samples *t*-test … comparing no-shift and shift conditions yielded substantial evidence against a main effect of condition on participants’ misinformation reliance in their inferential reasoning….” (p. 16), “Possible explanations for why Experiment 1 failed to find evidence for an effect of a spatial event boundary…” (p. 17), “A Bayesian independent-samples *t*-test provided substantial evidence against a difference between conditions in participants’ memory for the report….” (p. 25), and “There was substantial evidence against a main effect of condition on participants’ decision to charge a suspect….” (p. 26).

We also acknowledge that we do not have strong evidence to rule out a small effect (in the hypothesized direction) on p. 29, “Across both experiments, we failed to find evidence of a detrimental effect of a spatial event boundary on retraction integration, as reflected in similar inferential reasoning scores between no-shift and shift conditions in Experiment 1 and greater inference scores in the no-shift condition in Experiment 2. We acknowledge that while we can rule out a substantial effect, a small effect may be compatible with the observed results. However, the theoretical and practical relevance of such a small effect are questionable.”

References

[65] Brysbaert M. How Many Participants Do We Have to Include in Properly Powered Experiments? A Tutorial of Power Analysis with Reference Tables. J Cogn. 2019;2(1):1-38. <https://doi.org/10.5334/joc.72>

[66] Lakens D. Performing high‐powered studies efficiently with sequential analyses. Eur J Soc Psychol. 2014;44(7):701-10. <https://doi.org/10.1002/ejsp.2023>

[67] Schott E, Rhemtulla M, Byers-Heinlein K. Should I test more babies? Solutions for transparent data peeking. Infant Behav Dev. 2019;54:166-76. <https://doi.org/10.1016/j.infbeh.2018.09.010>
